# Recent studies of the molecular mechanism of lusitropy due to phosphorylation of cardiac troponin I by protein kinase A

**DOI:** 10.1007/s10974-022-09630-4

**Published:** 2022-09-21

**Authors:** Steven Marston

**Affiliations:** NHLI, Imperial College London, London, W12 ONN UK

**Keywords:** Ca^2+^ regulation, Phosphorylation, PKA, Troponin, Lusitropy, Adrenergic activation

## Abstract

Ca^2+^ acts on troponin and tropomyosin to switch the thin filament on and off, however in cardiac muscle a more graded form of regulation is essential to tailor cardiac output to the body’s needs. This is achieved by the action of adrenaline on β1 receptors of heart muscle cells leading to enhanced contractility, faster heart rate and faster relaxation (lusitropy) via activation of the cyclic AMP-dependent protein kinase, PKA. PKA phosphorylates serines 22 and 23 in the N-terminal peptide of cardiac troponin I. As a consequence the rate of Ca^2+^release from troponin is increased. This is the key determinant of lusitropy. The molecular mechanism of this process has remained unknown long after the mechanism of the troponin Ca^2+^ switch itself was defined. Investigation of this subtle process at the atomic level poses a challenge, since the change in Ca^2+^-sensitivity is only about twofold and key parts of the troponin modulation and regulation system are disordered and cannot be fully resolved by conventional structural approaches. We will review recent studies using molecular dynamics simulations together with functional, cryo-em and NMR techniques that have started to give us a precise picture of how phosphorylation of troponin I modulates the dynamics of troponin to produce the lusitropic effect.

John Squire played a major role in the deduction of the steric blocking mechanism of striated muscle regulation that was a seminal moment in muscle research (Parry and Squire, 1973). The model immediately seemed very plausible and it generated a huge effort in structural and biochemical investigations in muscle’s Ca^2+^-regulatory mechanism, an effort in which John played a significant role (Squire and Morris, 1998; Paul et al., 2009). Recently this work has culminated in high resolution cryo-em structures of the cardiac muscle thin filament that precisely located troponin and tropomyosin in the high and low Ca^2+^ states and that vindicate Squire’s original model (Yamada et al., 2020; Risi et al., 2021).

Ca^2+^ acts on troponin and tropomyosin to switch the thin filament on and off, however in cardiac muscle a more graded form of regulation is essential to tailor cardiac output to the body’s needs. This is achieved by the action of adrenaline on β1 receptors of heart muscle cells leading to enhanced contractility and faster heart rate.

The ‘flight or fight’ response to adrenaline has been known for around a century and its mechanism is well characterised. β1 receptor activation leads to cAMP production. cAMP acts directly on membrane channels and also activates the cyclic AMP-dependent protein kinase, PKA. PKA itself phosphorylates a variety of ion channels, ion pumps and contractile proteins. In the sarcomere PKA phosphorylates Myosin binding protein C (MyBP-C) and troponin I (TnI).

At the cellular and tissue level, activation of the cardiac muscle β1 receptors leads to a faster rate of contraction, an increased maximum twitch force and shortening and a faster rate of relaxation (see Fig. 1 (Wright et al., 2020)). This, combined with an increased heart rate leads to enhancement of cardiac output up to five-fold. The faster rate of relaxation is known as lusitropy; it is essential since it significantly shortens the heartbeat and thus enables efficient contraction at a higher heart rate.

**Fig. 1:**
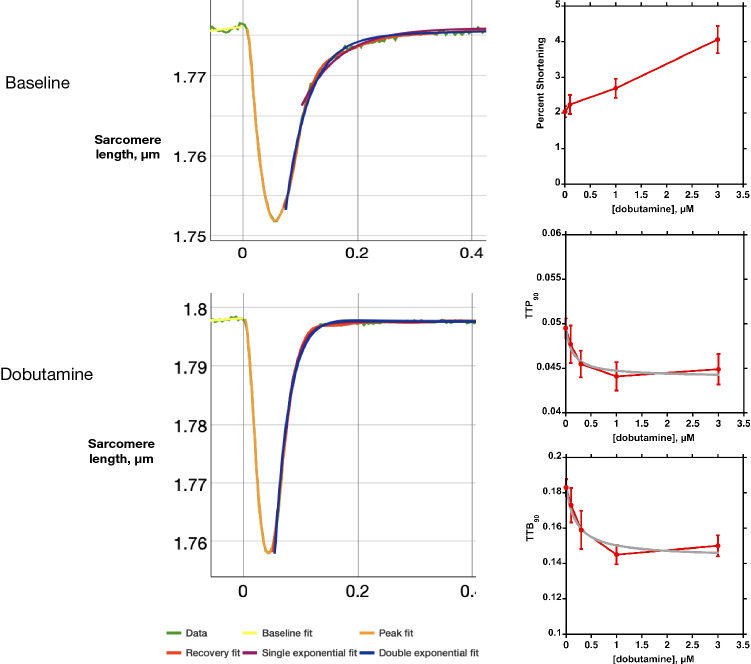
The effects of β1 receptor activation on cardiac myocyte contractility. Rat myocytes paced at 1 Hz, 37 °C. 1 µM dobutamine increases shortening amplitude and reduces contraction time (TTP90) and relaxation time (TTB90). Colours represent fitted parameters as described in the figure. Plots on the right show dose–response curves. Data from Wright et al. (2020)

It is well established that phosphorylation of troponin is essential for lusitropy (Ray and England, 1976; Solaro et al., 1976; Layland et al., 2005). Cardiac TnI has an N-terminal 33 amino acid peptide unique to the cardiac isoform. PKA phosphorylates serines 22 and 23 in this N-terminal peptide (NcTnI); as a consequence the rate of Ca^2+^ release from troponin C in diastole, is increased 2–threefold and recent studies indicate that at physiological temperatures Ca^2+^-dissociation can be rate-limiting for relaxation (Little et al., 2012). Since the Ca^2+^ dissociation constant = [rate Ca^2+^ off]/[rate Ca^2+^ on], phosphorylation of TnI also results in a reduced Ca^2+^-sensitivity of thin filament activation; consequently, the Ca^2+^ sensitivity parameter is often used to measure the effects of cTnI phosphorylation in vitro. A two to threefold change in Ca^2+^ sensitivity appears to be necessary and sufficient for lusitropy (Marston, 2016).

It is interesting to note that the response to adrenaline is of ancient origin, being present in bony fishes, but the lusitropic response and the phosphorylatable N terminal extension of TnI, only appear with air breathing quadrupeds (Fig. 2 (Rasmussen et al., 2022)). Presumably the demand of life on land require a faster heart rate than life in water, making lusitropy necessary.

**Fig. 2:**
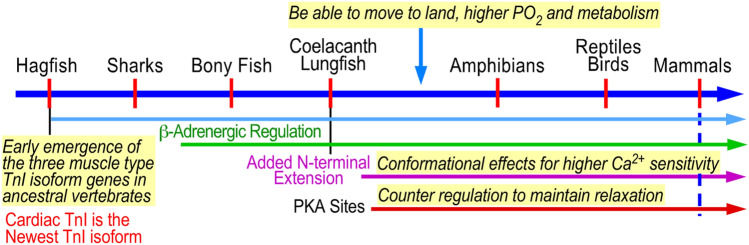
Evolutionary timeline for the development of the N-terminal phosphorylatable peptide of cTnI and lusitropy. Figure kindly provided by Dr J-P Lin, Univ Illinois

The essential role of cTnI phosphorylation for lusitropy has been demonstrated in transgenic mice where the Serines 22 and 23 are mutated to Alanine (Pi et al., 2002) or aspartic acid (Yasuda et al., 2007) or where there is a cardiomyopathy mutation that uncouples TnI phosphorylation from the change in Ca^2+^ sensitivity (Song et al., 2011; Wilkinson et al., 2015). In fact, many, if not all, the mutations in thin filament proteins that have been found to cause cardiomyopathy (Hypertrophic or dilated cardiomyopathy) have been found to be uncoupled (Messer and Marston, 2014). In the case of dilated cardiomyopathy, uncoupling is the only abnormality common to all thin filament mutations. Moreover in at least one case, the ACTC E361G mutation causing DCM, uncoupling was the only abnormality detected and this abnormality was sufficient to induce symptoms of heart failure in mutant mice, but only under stress (Wilkinson et al., 2015).

Restoring lusitropy is thus an attractive target for drug treatments in cases of inherited cardiomyopathy. EGCG, Silybin B and some of their derivatives have been found to be very effective as recouplers (Sheehan et al., 2018). Many of the compounds currently studied have a multiplicity of pharmacological actions (Křen and Valentová, 2022) but Silybin B and Resveratrol appear to be pure recouplers in vitro and in cardiomyocytes, worthy of further investigation (Fig. 3). It is noteworthy that a recent study of EGCG has shown it to be effective in relieving DCM induced by pressure overload (Mou et al., 2022).

**Fig. 3:**
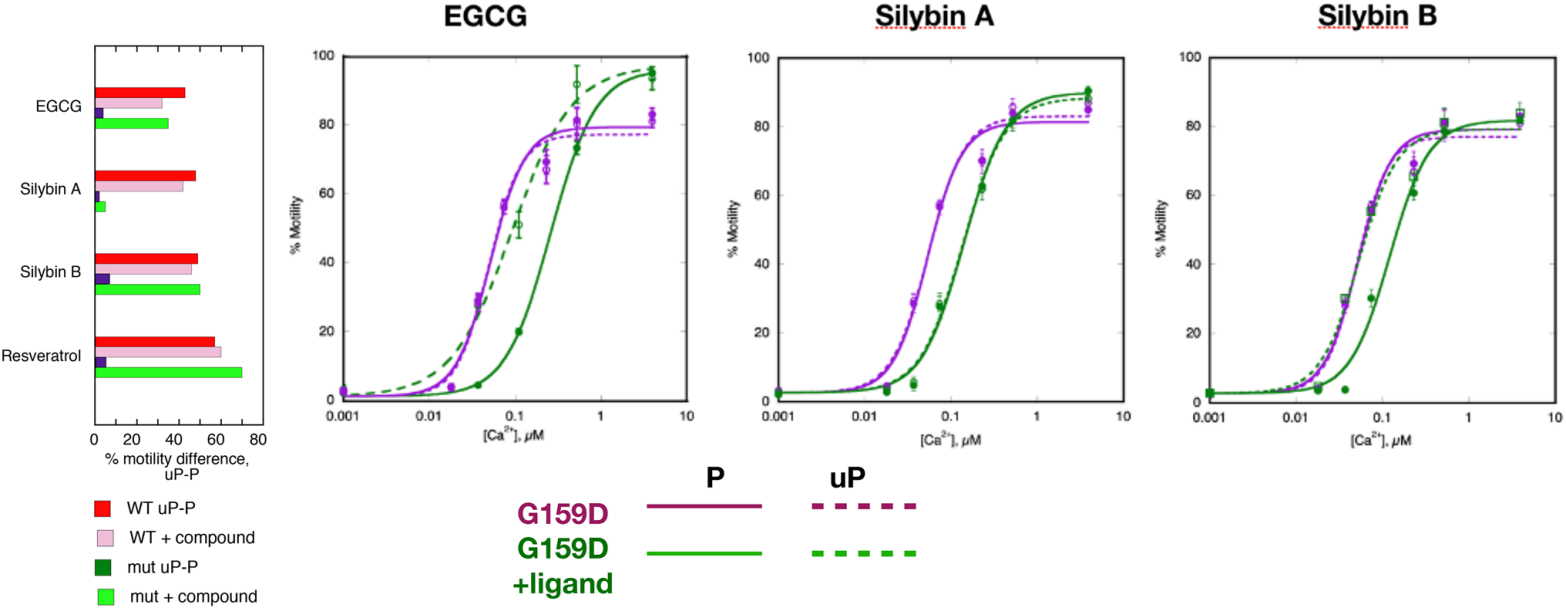
Small molecules that can restore the response to phosphorylation in mutant thin filaments. Measurement made by in vitro motility assay in reconstituted human thin filaments. Left: fixed [Ca^2+^] assay (~ ec_50_) using WT or TPM1 E180G HCM mutant. Small molecules do not affect wild-type but EGCG, Silybin B and resveratrol; restore the phosphorylation effect whilst Silybin A does not. The same effect of the small molecules is shown by the measurements of the Ca^2+^-activation curve for phosphorylated and unphosphorylated DCM mutant (TNNC1 G159D) thin filaments

The molecular mechanism of this process has remained unknown long after the mechanism of the troponin Ca^2+^ switch itself was defined (Li et al., 1999; Takeda et al., 2003; Vinogradova et al., 2005). Investigation of this subtle process at the atomic level poses a challenge for structural biology since the change in Ca^2+^-sensitivity is only about twofold (Marston, 2016) and key parts of the troponin modulation and regulation system are disordered (Kowlessur and Tobacman, 2012) and cannot be fully resolved. (Fig. 4).

**Fig. 4:**
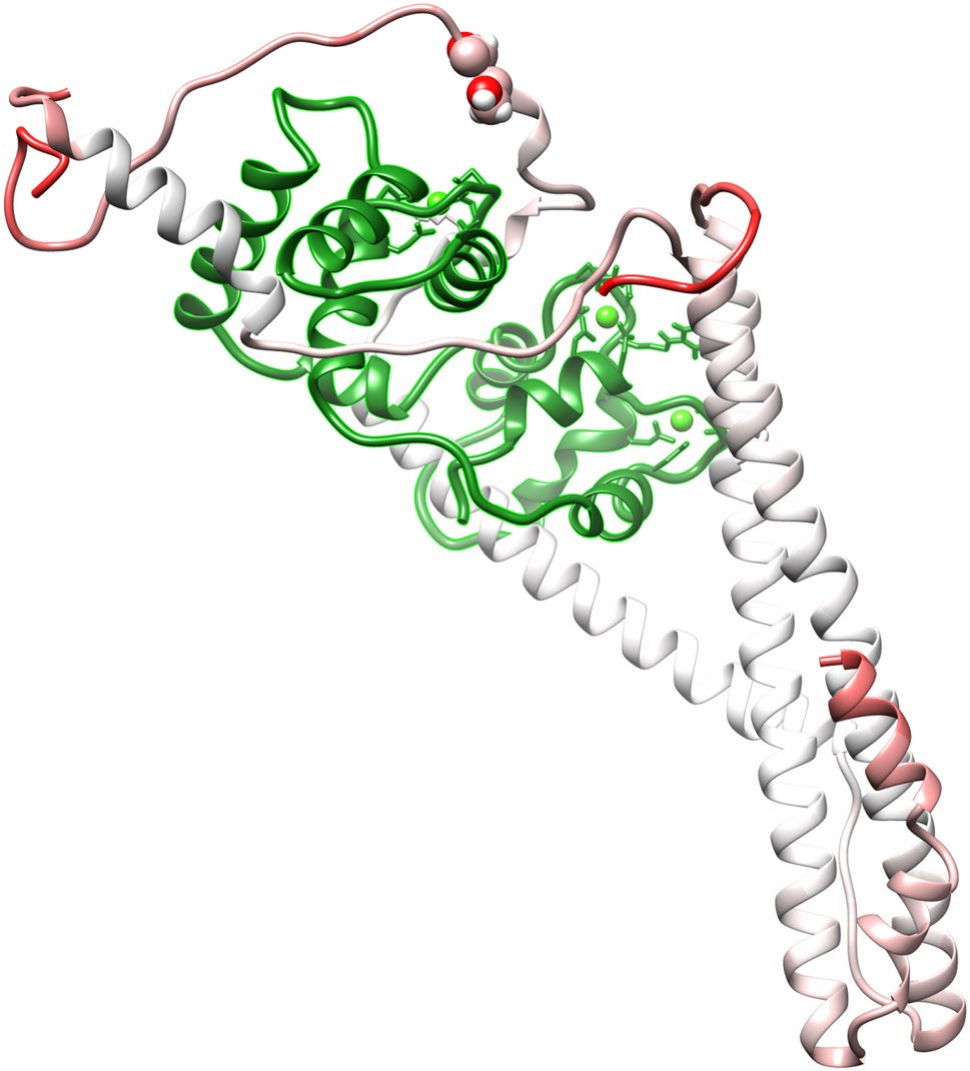
Structural representation of the backbone for the most populated state of the unphosphorylated troponin core, determined by molecular dynamics simulation. The peptide chains are coloured according to their RMSF, with the highest RMSF deepest red. The most mobile areas are NcTnI (1–32), CcTnT (278–288) and cTnI “inhibitory” peptide (137–148). This result is very similar to that measured by H–D Exchange (Kowlessur and Tobacman, 2012). Serines 22 and 23 represented as spheres. TnC is coloured green

To understand the mechanism of regulation by troponin I phosphorylation and the effects of mutations, methods that can describe the dynamics of troponin such as NMR techniques (Hwang et al., 2014; Matsuo et al., 2015; Mahmud et al., 2021) have been employed but they have their limitations since only incomplete peptides of troponin are studied. The first attempt to model the NcTnI peptide docked onto the N-terminal troponin was by Howarth et al. (Howarth et al., 2007). This work indicated that the phosphorylated and unphosphorylated forms bound differently and proposed that phosphorylation weakened NcTnI interactions with the N-lobe of cTnC and re-positioned the acidic amino terminus of cTnI1-32 for favourable interactions with basic regions. This model may or may not be correct but it has been used as a starting point for further modelling of whole troponin. A seminal NMR study by Baryshnokova et al. (Baryshnikova et al., 2008) titrated NcTnI and switch peptides binding to NcTnC. It established that the NcTnI and the switch region of cTnI bind to cNTnC simultaneously. The binding energy is barely affected by phosphorylation but the signal is transferred from the cTnI N-terminus, increasing the cNTnC affinity toward the switch peptide twofold (K_d_ of switch peptide to NcTnC binding decreased from 600 to 370 µM on phosphorylation) but not affecting Ca^2+^ affinity, thus the concept of a conformational relay between the TnI phosphorylation site and the switch peptide was established.

Two recent papers have advanced our understanding of this process. Using solution NMR ^15 ^N R2 relaxation rate analysis, Mahmud et al. (2021) demonstrated that the N- and C-terminal domains of cTnC tumble independently, being connected by a flexible linker, but upon addition of cTnI1-77 (includes NcTnI and Helix 1), the complex tumbles as a rigid rod (Fig. 5). The cTnI phosphomimetic mutants S22D/S23D and also DCM-associated mutations including cTnC G159D partially destabilize this ‘active’ conformation of cNTnC. The authors propose that phosphorylation and mutations modulate the degree of inter-domain tethering and release and could account for the modulation of Ca^2+^ sensitivity and Ca^2+^ release rate. This study and its predecessors indicate the important role of the NcTnC-CcTnC interdomain interface in lusitropic regulation.

**Fig. 5:**
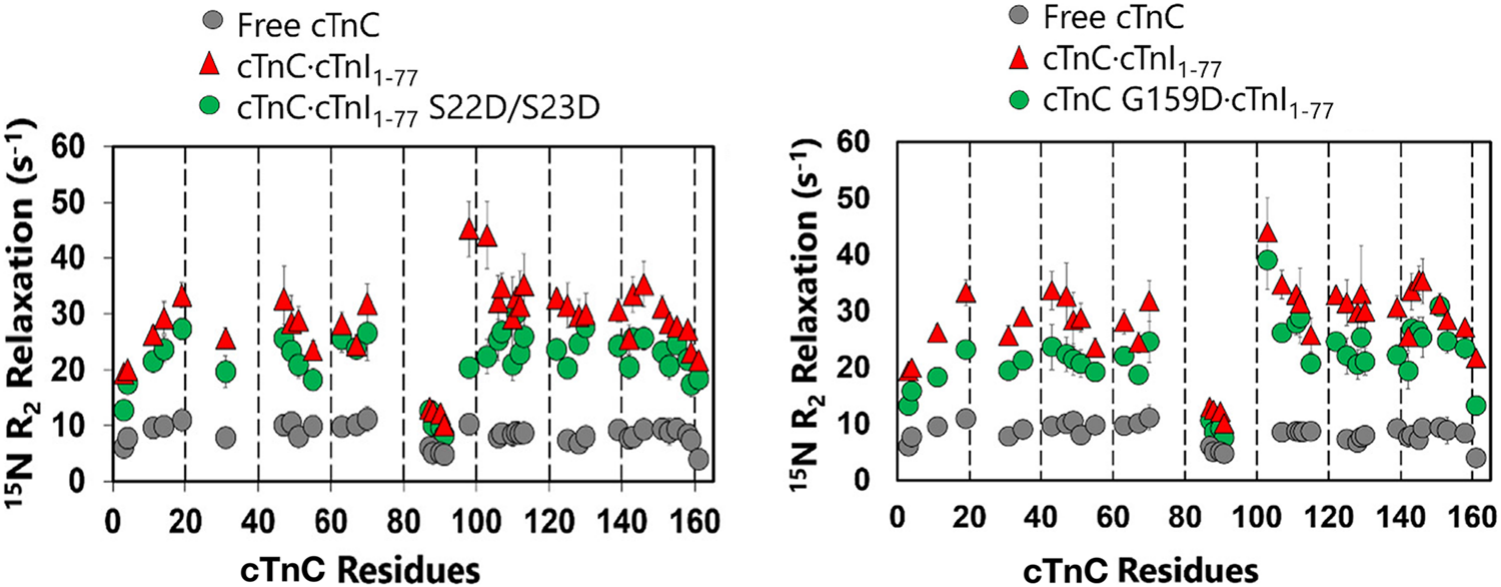
cTnC backbone ^15^ N R_2_ relaxation time measurements for TnC-TnI peptide complexes. Plots of ^15^ N R_2_ relaxation time vs cTnC residue number for free cTnC and complexes with cTnI 1–77 Left: comparison of ^15^ N R_2_ relaxation rates of free cTnC, cTnC complexed with wildtype cTnI1-77, and cTnC complexed with phosphomimetic cTnI1-77 S22/23D. Right: Comparison of ^15^ N R_2_ relaxation rates of free cTnC, cTnC complexed with wildtype cTnI1-77, and substituted DCM mutant cTnC G159D. Figure kindly supplied by Dr P Hwang, Univ Alberta

A recent paper from Kachooei et al. (2021) provides a further insight into the structural and dynamic effects of troponin I phosphorylation with the advantage that the measurements are made on whole troponin rather than selected peptides. They used a paramagnetic spin labelling approach to position and track the movement of the NcTnI region within whole Tn. Paramagnetic relaxation enhancement (PRE)-NMR experiments, showed that the NcTnI region interacts with a broad surface area on NcTnC. Phosphorylation of NcTnI both weakens and shifts this region to an adjacent site on NcTnC. Interspin EPR distances between NcTnI and NcTnC further reveal a phosphorylation-induced re-orientation of the TnC N-domain relative to the ITC domain under saturating Ca^2+^conditions. This work, therefore, confirms the connection between phosphorylation of NcTnI, NcTnC rearrangement and cTnC interdomain interactions. The authors propose an allosteric model where phosphorylation triggers cooperative changes in both the interaction of the NcTnI region with TnC, and the TnC interdomain orientation, together this would promote the release of the TnI switch-peptide leading to enhancement of the myocardial relaxation rate,

Recently computational molecular modelling and all-atom molecular dynamics simulations (MD) have become the method of choice for understanding the phosphorylation-dependent modulation the cardiac troponin. (Cheng et al., 2014; Papadaki and Marston, 2016; Zamora et al., 2016). In principal MD can predict troponin structure and dynamics but there are practical constraints on this methodology. The technique calculates atomic structure from first principles and this depends on having an adequate set of force field parameters to work with. This seems to have been achieved now so the main constraints are linked to computing capacity.

The first studies suffered from using a small virtual box to contain troponin, which is problematical due to the L-shape of troponin that may reach the edge of the box and create artefactual virtual intermolecular interactions. Recent studies use a 140 Å cube. Because key parts of troponin are disordered, it is also necessary to ensure that the time dependence of the calculations represent a steady state rather than an approach to an equilibrium. To be sure, long simulations must be run. Recent studies used 5 × 1500 ns runs and were able to demonstrate that the fluctuations observed were simply time-independent stochastic changes of the disordered NcTnI, CcTnT the ‘inhibitory peptide of TnI and the cTnC interdomain linker (Zamora, 2019; Yang et al., 2021). The rest of the structure closely resembled the structure derived from X-ray diffraction (Takeda et al., 2003).

The effects of phosphorylation of NcTnI and of disease-causing mutations cannot be described by any static structure, but rather by a shift in the populations of a number of rapidly interchanging states. In our recent studies this has been characterised by the Arpeggio technique which calculates the probabilities of all possible interactions in every frame (37,500) of the simulation (Jubb et al., 2017). This analysis has revealed that the direct effect of phosphorylation at serines 22 and 23 is a local rearrangement (Fig. 6). The major interaction between NcTnC and NcTnI is TnC Asp33, in the EF hand loop I, with TnI Arg 1920 and 21. This is significant as Asp 33 is one of the few cardiac-specific variants in cTnC (Gly33 in skeletal muscle TnC). Upon phosphorylation, there is a significant cumulative loss of these interactions from 84 to 19%.

**Fig. 6:**
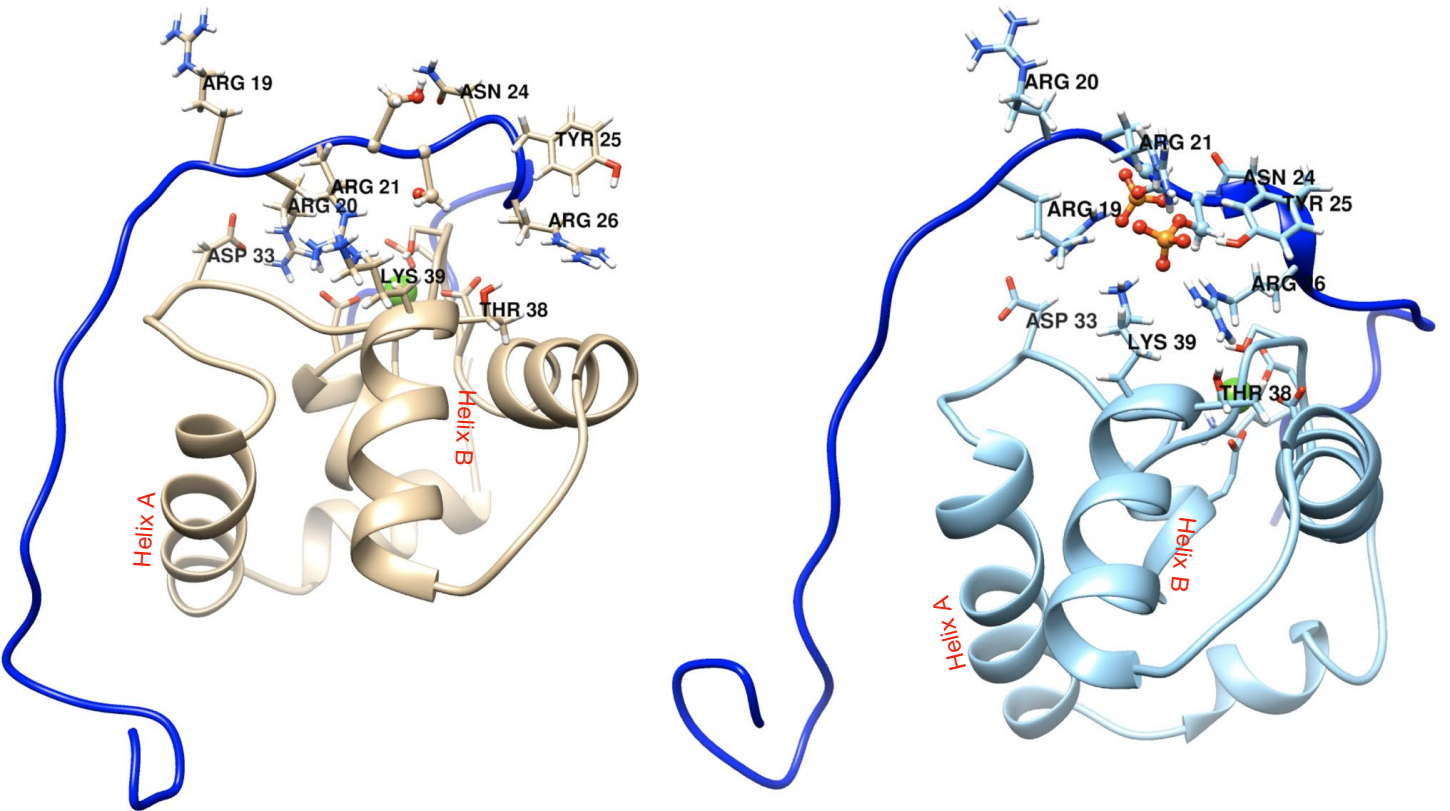
Wild Type molecular dynamics representative structure showing NcTnC and NcTnI only in unphosphorylated and phosphorylated states. The major phosphorylation-dependent interactions between NcTnC and NcTnI side chains are shown. NcTnI (1–34) is shown in blue, NcTnC is shown in brown (unphosphorylated) and pale blue (phosphorylated). Helix A and B are indicated. Figure kindly supplied by Zeyu Yang

Associated with this, several basic amino acids, including arginines in NcTnI (Arg19, 21 and 26) and cTnC lysine 39 tend to form a cluster of ionic bonds with the phosphate groups of phosphoserines 22 and 23. (NcTnI Arg19, 21 and 26 and NcTnC Lys39 interactions with phosphoserine increase to 68%, 72%, 95% and 66% respectively from 0% in the unphosphorylated case).

Consequential upon this phosphorylation-induced change, the pulling of Lys 39 upwards impinges upon the helix B and consequently its orientation relative to Helix A and the hydrophobic patch that the switch peptide binds to, manifested as the change in mean helix A/B interhelical angle. Longer range allosteric consequences of the helix B rearrangement include a repositioning of the switch peptide and changes in the peptides crossing the interdomain interface that can account for the observed changes in mean interdomain hinge angle.

As a reality check, the G159D mutation in troponin C that causes dilated cardiomyopathy and also uncouples phosphorylation from the Ca^2+^-sensitivity change was studied (Biesiadecki et al., 2007; Dyer et al., 2009). The primary difference due to the G159D mutation is the formation of strong and phosphorylation-independent bonds across the bottom of the interdomain interface, particularly the probability of interaction between cTnC Arg 83 and the mutated Asp159; consequently, the range of hinge angles is reduced by this constraint. These interactions may also relocate the apparent fulcrum of the interdomain ‘hinge’ so that the phosphorylation-dependent changes in the peptides crossing the interdomain interface can result in opposite changes in interdomain angle consistent with the functional abnormalities caused by the mutation (Fig. 7).

**Fig. 7:**
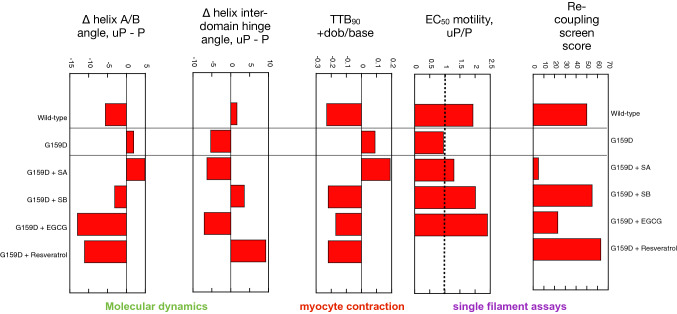
Comparison of the effect of phosphorylation of cardiac troponin on its biochemical, physiological and molecular dynamics parameters, its suppression by the TnC G159D DCM related mutation and its restoration by small molecules. The response to phosphorylation measured by an in vitro motility screen at fixed Ca^2+^ (data from Sheehan et al. 2018). Measurement of the change in thin filament Ca.^2+^ sensitivity on phosphorylation expressed a EC_50_ uP/P ratio, measured by motility assay (data from Sheehan et al. 2018). Lusitropic effect measured in wild-type or mutant cardiomyocytes paced at 1 Hz, 37 °C expressed as the fractional change in ttb_90_ due to dobutamine (see Fig. 1; data from Sheehan, 2019). Change in the A/B and interdomain hinge angles on phosphorylation calculated by molecular dynamics simulations are shown (data from Yang et al 2021). SA, SilybinA; SB, SilybinB; EGCG, epigallocatechin-3 gallate. DCM mutations block the response to phosphorylation but it can be restored by Silybin B, EGCG and Resveratrol (Yang et al. 2021)

Overall, these changes in dynamics are compatible with the NMR studies, described above, and extend our understanding of the linking of phosphorylation to Ca^2+^-sensitivity and release rate to the atomic level. As a further test of our molecular dynamics analysis we Simulated the effects of small molecules that can restore coupling to mutations (See Fig. 3) (Sheehan et al., 2018); in fact, we have found that the pure recouplers, SilybinB and resveratrol can revert the phosphorylation-dependent changes in G159D helix A/B and interdomain angle distributions to that of wild type whilst the inactive Silybin A does not (Zamora, 2019; Yang et al., 2021).

Finally, it should be understood that, in the muscle thin filament, troponin is associated with actin and tropomyosin, therefore studies based on the troponin core only are incomplete. Cryo-EM studies of the thin filament have precisely located troponin in relation to tropomyosin and actin in the absence and presence of bound Ca^2+^ (Yamada et al., 2020; Risi et al., 2021). These structures do not include the disordered parts of troponin but molecular dynamics simulations can indicate a likely location for the phosphorylatable N-terminal peptide of TnI. Preliminary results indicate that it could be close to tropomyosin and form phosphorylation-dependent interactions that have not been accounted for in studies of the isolated troponin core (Pavadai et al., 2022) (Fig. 8).

**Fig. 8:**
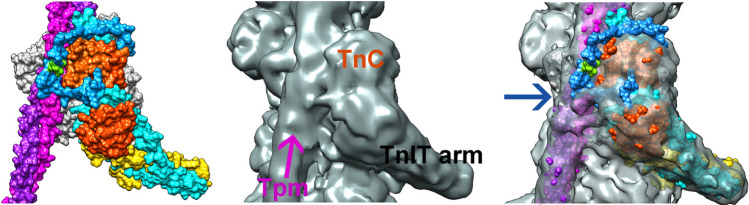
Position of the cardiac N-terminal TnI domain in the thin filament model. (Left) Initial model showing the troponin core-domain and the N-terminal extension of cardiac TnI abutting actin and tropomyosin (one actin subunit shown, grey; tropomyosin, magenta/purple; TnC, orange; TnT, yellow; TnI residues 41–171, cyan; and N-terminal TnI residues 1–40, blue with its Ser23/24 residues coloured green). (middle) Surface rendering of the central segment of the Yamada et al. cryo-EM reconstruction of cardiac thin filaments (right) The thin filament model superimposed on the cryo-EM thin filament volume in made translucent. Figure kindly provided by Dr W Lehman, Boston University

These simulations did not include the other disordered parts of troponin such as CcTnT, so further studies may uncover different interactions between troponin, tropomyosin and actin involved in the response to NcTnI phosphorylation at serines 22 and 23.

In conclusion, molecular dynamics simulations together with functional, cryo-em and NMR techniques have started to give us a better picture of how phosphorylation of troponin I modulates the dynamics of troponin to produce the lusitropic effect. Remarkably, as summarised in Fig. 7, the changes induced by phosphorylation, mutation and small molecules are all consistent between various measurement techniques from the atomic to the cellular level, indicating the likely validity of the proposed mechanism of lusitropy.

There is still much more to be found out about this system using these new tools.
